# Personality Polygenes, Positive Affect, and Life Satisfaction

**DOI:** 10.1017/thg.2016.65

**Published:** 2016-08-22

**Authors:** Alexander Weiss, Bart M. L. Baselmans, Edith Hofer, Jingyun Yang, Aysu Okbay, Penelope A. Lind, Mike B. Miller, Ilja M. Nolte, Wei Zhao, Saskia P. Hagenaars, Jouke-Jan Hottenga, Lindsay K. Matteson, Harold Snieder, Jessica D. Faul, Catharina A. Hartman, Patricia A. Boyle, Henning Tiemeier, Miriam A. Mosing, Alison Pattie, Gail Davies, David C. Liewald, Reinhold Schmidt, Philip L. De Jager, Andrew C. Heath, Markus Jokela, John M. Starr, Albertine J. Oldehinkel, Magnus Johannesson, David Cesarini, Albert Hofman, Sarah E. Harris, Jennifer A. Smith, Liisa Keltikangas-Järvinen, Laura Pulkki-Råback, Helena Schmidt, Jacqui Smith, William G. Iacono, Matt McGue, David A. Bennett, Nancy L. Pedersen, Patrik K. E. Magnusson, Ian J. Deary, Nicholas G. Martin, Dorret I. Boomsma, Meike Bartels, Michelle Luciano

**Affiliations:** 1 Centre for Cognitive Ageing and Cognitive Epidemiology, Department of Psychology, School of Philosophy, Psychology and Language Sciences, The University of Edinburgh, Edinburgh, UK; 2 Department of Biological Psychology, Netherlands Twin Register, VU University Amsterdam, Amsterdam, the Netherlands; 3 EMGO+ Institute for Health and Care Research, VU University Medical Centre, Amsterdam, the Netherlands; 4 Clinical Division of Neurogeriatrics, Department of Neurology, Medical University Graz, Austria; 5 Institute of Medical Informatics, Statistics and Documentation, Medical University Graz, Austria; 6 Rush Alzheimer’s Disease Center, Rush University Medical Center, Chicago, IL, USA; 7 Department of Neurological Sciences, Rush University Medical Center, Chicago, IL, USA; 8 Department of Applied Economics, Erasmus School of Economics, Erasmus University Rotterdam, Rotterdam, the Netherlands; 9 Department of Epidemiology, Erasmus Medical Center, Rotterdam, the Netherlands; 10 Erasmus University Rotterdam Institute for Behavior and Biology, Rotterdam, the Netherlands; 11 Quantitative Genetics, QIMR Berghofer Institute of Medical Research, Brisbane, Queensland, Australia; 12 Department of Psychology, University of Minnesota, USA; 13 Department of Epidemiology, University of Groningen, Groningen, the Netherlands; 14 Survey Research Center, Institute for Social Research, University of Michigan, Ann Arbor, MI, USA; 15 Division of Psychiatry, University of Edinburgh, Royal Edinburgh Hospital, Edinburgh, UK; 16 Department of Epidemiology, School of Public Health, University of Michigan, Ann Arbor, MI, USA; 17 Interdisciplinary Center Psychopathology and Emotion regulation, University Medical Center, University of Groningen, Groningen, the Netherlands; 18 Department of Behavioral Sciences, Rush University Medical Center, Chicago, Illinois, USA; 19 Department of Psychiatry, Erasmus Medical Center, Rotterdam, the Netherlands; 20 Department of Child and Adolescent Psychiatry, Erasmus Medical Center, Rotterdam, the Netherlands; 21 Department of Neuroscience, Karolinska Institutet, Stockholm, Sweden; 22 Department of Medical Epidemiology and Biostatistics, Karolinska Institutet, Stockholm, Sweden; 23 Program in Translational NeuroPsychiatric Genomics, Institute for the Neurosciences, Departments of Neurology and Psychiatry, Brigham and Women’s Hospital, Boston, MA, USA; 24 Harvard Medical School, Boston, MA, USA; 25 Program in Medical and Population Genetics, Broad Institute, Cambridge, MA, USA; 26 Division of Biology and Biomedical Sciences, Washington University, MO, USA; 27 Institute of Behavioural Sciences, University of Helsinki, Finland; 28 Geriatric Medicine Unit, Western General Hospital, Edinburgh, and Centre for Cognitive Ageing and Cognitive Epidemiology, University of Edinburgh, UK; 29 Department of Economics, Stockholm School of Economics, Stockholm, Sweden; 30 Department of Economics, New York University, New York, USA; 31 Research Institute for Industrial Economics, Stockholm, Sweden; 32 Medical Genetics Section, University of Edinburgh Centre for Genomic and Experimental Medicine and MRC Institute of Genetics and Molecular Medicine, Western General Hospital, Crewe Road, Edinburgh, UK; 33 IBS, Unit of Personality, Work and Health, Institute of Behavioural Sciences, University of Helsinki, Finland; 34 Helsinki Collegium for Advanced Studies, University of Helsinki, Finland; 35 Department of Neurology, Medical University Graz, Austria; 36 Institute of Molecular Biology and Biochemistry, Centre for Molecular Medicine, Medical University of Graz, Graz, Austria; 37 Department of Psychology, University of Michigan, Ann Arbor, MI, USA; 38 Neuroscience Campus Amsterdam, Amsterdam, the Netherlands

**Keywords:** wellbeing, genetics, polygenic prediction, happiness, genetic correlation

## Abstract

Approximately half of the variation in wellbeing measures overlaps with variation in personality traits. Studies of non-human primate pedigrees and human twins suggest that this is due to common genetic influences. We tested whether personality polygenic scores for the NEO Five-Factor Inventory (NEO-FFI) domains and for item response theory (IRT) derived extraversion and neuroticism scores predict variance in wellbeing measures. Polygenic scores were based on published genome-wide association (GWA) results in over 17,000 individuals for the NEO-FFI and in over 63,000 for the IRT extraversion and neuroticism traits. The NEO-FFI polygenic scores were used to predict life satisfaction in 7 cohorts, positive affect in 12 cohorts, and general wellbeing in 1 cohort (maximal *N* = 46,508). Meta-analysis of these results showed no significant association between NEO-FFI personality polygenic scores and the wellbeing measures. IRT extraversion and neuroticism polygenic scores were used to predict life satisfaction and positive affect in almost 37,000 individuals from UK Biobank. Significant positive associations (effect sizes <0.05%) were observed between the extraversion polygenic score and wellbeing measures, and a negative association was observed between the polygenic neuroticism score and life satisfaction. Furthermore, using GWA data, genetic correlations of −0.49 and −0.55 were estimated between neuroticism with life satisfaction and positive affect, respectively. The moderate genetic correlation between neuroticism and wellbeing is in line with twin research showing that genetic influences on wellbeing are also shared with other independent personality domains.

Happiness is a desirable state that is universally pursued. It is also linked to personality traits, such as those of the Five-Factor Model (Adams et al., 2012; DeNeve & Cooper, 1998). Individuals who score lower on neuroticism and higher on extraversion, agreeableness, and conscientiousness report being happier and more satisfied with their lives (meta-analytic correlations ranged 0.17–0.22; DeNeve & Cooper, 1998). Genetic influences account for approximately 40% of variation in wellbeing (Bartels, 2015), which is comparable to the heritability estimates for personality traits (Bouchard & Loehlin, 2001). Genetic analysis has shown that although unique, non-additive genetic effects were found for happiness and general quality of life (Bartels & Boomsma, 2009), a common additive genetic factor influences different well-being measures (i.e., general quality of life, present quality of life, life satisfaction, and subjective happiness/positive affect).

Evidence for shared genetic variance between personality and wellbeing comes from biometric genetic studies of great ape pedigrees (Adams et al., 2012; Weiss et al., 2002). It also comes from studies of human twins and siblings. Using a three-item wellbeing measure (present and general life satisfaction, control over one’s life), Weiss et al. (2008) showed that a general personality additive genetic factor explained 2.2% of the variance in wellbeing. Additional genetic contributions to wellbeing were via independent factors that influenced neuroticism (5.3% of variance), extraversion (13%), and conscientiousness (0.8%). Hahn et al. (2013) confirmed the absence of unique genes influencing a multidimensional measure of life satisfaction in their extended twin study, additionally showing shared non-additive genetic variance between neuroticism and life satisfaction.

A complementary test of the hypothesis that common genes underlie variation in personality and happiness is to use molecular data, such as single nucleotide polymorphisms (SNPs). In a recent large study (*N* ≈ 300K), a polygenic score constructed from a genome-wide association (GWA) meta-analysis on subjective wellbeing explained ~0.7% of the variance in neuroticism and ∼0.4% of the variance in extraversion (Okbay et al., 2016). Applying bivariate linkage disequilibrium score regression (Bulik-Sullivan et al., 2015) to the GWA summary statistics for wellbeing and neuroticism resulted in a SNP-based genetic correlation of −0.75 (*SE* = 0.034; Okbay et al., 2016). This genetic correlation represents the correlation of common, additive genetic effects between the two traits. Whereas the variance in a trait explained by polygenic scores is typically low, methods to infer the expected SNP-derived variance from polygenic scores show agreement with their empirical and simulation-based estimates (Dudbridge, 2013; Wray et al., 2014).

To provide greater support for a genetic association between personality and wellbeing, our aim here is to predict phenotypic scores for wellbeing and its subcomponents of life satisfaction and positive affect by using information about SNP effects on neuroticism, extraversion, openness, agreeableness, and conscientiousness. We used a method involving polygenic prediction models that enabled us to test whether genes influencing one trait influence another trait (for a review, see Wray et al., 2014). In this method, GWA results of a trait are used to create a polygenic score representing the sum of the effects of individual SNPs on that trait in an independent sample. This score is then used to predict the trait of interest. Polygenic prediction models do not require family designs, enabling the use of a large number of population-based studies with wellbeing and genotyping data.

We furthermore established genetic correlations between neuroticism and wellbeing measures by using a bivariate restricted maximum likelihood (REML) estimation (Lee et al., 2012) that has not previously been applied to these traits. This method uses genome-wide SNP data to calculate a genetic relationship matrix between unrelated individuals which within a REML framework allows estimation of the heritability due to all SNPs. This extends to the bivariate case from which genetic correlations can be ascertained.

We created polygenic scores using GWA results for the NEO Five-Factor Inventory (NEO-FFI; de Moor et al., 2012) and for extraversion and neuroticism from item response theory (IRT) analyses of varying personality scales (de Moor et al., 2015; van den Berg et al., 2015). Whereas the NEO-FFI GWA meta-analysis comprised a smaller total sample size (*N* = 17,375) than the IRT extraversion and neuroticism GWA meta-analyses (*N* ~ 63,000), importantly, it measures all five personality domains, and polygenic prediction based on these results has been successful for extraversion (predicting bipolar disorder) and neuroticism (predicting major depressive disorder; Middeldorp et al., 2011). We used unit-weighted tests to determine whether the polygenic score of any personality domain was associated with phenotypic variance in life satisfaction, positive affect, and wellbeing. For the NEO-FFI GWA results, polygenic prediction was tested in 14 cohorts that were independent of the GWA, and for the IRT extraversion and neuroticism GWA results, polygenic prediction was tested in the UK Biobank, which was independent of the GWA meta-analyses. To establish genetic correlations between neuroticism and wellbeing using bivariate REML, we used a large cohort of unrelated individuals with genome-wide data and measurements on all the traits of interest.

## Methods

### Participants

#### NEO-FFI polygenic prediction in 14 cohorts

Cohorts were drawn from a GWA study meta-analysis of wellbeing conducted by the Social Sciences Genetic Association Consortium (SSGAC; http://www.thessgac.org), with the proviso that none of the cohorts were part of the GWAS meta-analysis of the NEO-FFI (de Moor et al., 2012); personality data were not required for analysis. Participants were (or were ancestors of) white Europeans. Thirteen cohorts with positive affect (*n* ranged 351–11,971) and seven cohorts with life satisfaction (*n* ranged 351–9,938) were available (five cohorts had positive affect and life satisfaction measures) for inclusion in our meta-analysis. An additional cohort (*n* = 6,960) had a measure of general wellbeing that was analyzed separately. Individual cohort descriptions, including the scales and/or items used to measure wellbeing, are provided in the Supplementary information. The relevant institutional ethics review boards approved the individual studies.

DNA was extracted using standard protocols. Genotyping procedures are summarized in Supplementary Table S1. Cohorts used HapMap II imputed data or, if unavailable, observed genotypes for analysis. Imputed data were preferred because the GWAS personality results were based on HapMap II data, thus ensuring that all SNPs would be matched to those available in the GWAS. One cohort used 1000G imputation but removed SNPs that were not available in HapMap II.

#### Extraversion and neuroticism polygenic prediction in UK Biobank

Five of the SSGAC cohorts participated in the IRT extraversion and neuroticism GWA studies (de Moor et al., 2015; van den Berg et al., 2015); therefore, another independent cohort was sought for this prediction analysis. Participants were drawn from the baseline survey of the UK Biobank (http://www.ukbiobank.ac.uk), a resource established for investigating factors influencing disease in middle and older age. These measures (including questionnaire and biological samples) were collected between 2006 and 2010 on 502,655 British community residing individuals, a subset of whom were used in the present study. Positive affect was measured by the item ‘In general how happy are you?’ on a six-point scale (*extremely happy, very happy, moderately happy, moderately unhappy, very unhappy*, and *extremely unhappy*). General life satisfaction was surveyed across family relationships, financial situation, friendship, health, and work/job domains on the same six-point scale. Responses on these items demonstrated positive manifold and were best described by a single factor that explained 37% of variance. An averaged life satisfaction score was used to account for missing data where a person was currently unemployed (*n* = 11,679), did not know (*n* ranged 97–380), or preferred not to answer (*n* ranged 30–170). Neuroticism was measured by 12 items from the Eysenck Personality Questionnaire Revised (Eysenck & Eysenck, 1991). Wellbeing data were available for 36,737 (positive affect) and 36,911 (life satisfaction) individuals with genome-wide genotyping data. These data were skewed in the direction of lower positive affect/life satisfaction, but no ceiling effect was present. Ages ranged between 40 and 70 years (mean age = 57.31 years, *SD* = 7.92).

DNA was obtained via blood samples and genotyping performed with either the UK BiLEVE array or the UK Biobank axiom array. Standard quality control procedures were followed, including checks for gender mismatch and non-British ancestry. Further description can be found in Hagenaars et al. (2016). Polygenic scores were created on the observed genotypes. UK Biobank received ethical approval from the Research Ethics Committee (REC reference 11/NW/0382).

### Statistical Analysis

#### NEO-FFI polygenic prediction in 14 cohorts

Five sets of polygenic scores representing the personality domains of neuroticism, extraversion, openness, agreeableness, and conscientiousness were estimated using SNP association results from the largest GWA meta-analysis of NEO-FFI domains to date (de Moor et al., 2012). This GWA study included 10 discovery samples (*N* = 17,375). None of the cohorts – except NTR – in the present study were part of this personality GWA. For their analyses, NTR removed the participants who were part of the personality GWA meta-analysis.

Personality polygenic scores were estimated in each cohort using five probability thresholds for choosing SNPs to include in the score. These were based on the significance value for each SNP from the GWAS meta-analysis: *p* < .01, *p* < .05, *p* < .1, *p* < .5, and *p* < 1. Polygenic scores were formed by summing the meta-analytic effect size coefficients (betas) weighted by the number of copies (0/1/2) of the effect allele carried by the individual across all SNPs within the threshold. For imputed data, best guess genotypes were used but excluding SNPs with an imputation quality estimated *r*^2^ less than 0.80. Before score calculation, SNPs with a minor allele frequency <0.05 and Hardy–Weinberg Equilibrium test *<p* × 10^−7^ were removed. SNPs were then pruned for linkage disequilibrium using an *r*^2^ cut-off of 0.25 within a 200-SNP sliding window, following Purcell et al. (2009). Missing SNPs for an individual were imputed dependent on the observed allele frequency in the cohort. Polygenic scores were calculated using PLINK (Purcell et al., 2007). Supplementary Table S2 shows the number of SNPs included in the calculation of the polygenic score at each threshold for all the cohorts.

To predict phenotypic wellbeing scores from the polygenic personality scores, regression analysis was used. The dependent measures (positive affect, life satisfaction, and general wellbeing) were residualized on age, age squared (if significant), sex, population stratification components, and number of non-missing SNPs contributing to each individual’s score (where observed genotypes were used or where sparse genotyping led to poorer imputation quality). Standardized residual scores were then used as the dependent variable. A series of univariate regression analyses using each of the five polygenic personality scores as predictors was run for each polygenic score threshold (i.e., 25 tests). For the MCTFR cohort, a feasible generalized least squares regression was used to account for familial correlations. For NTR, a generalized estimating equating model was used to account for family structure. A meta-analysis of the standardized regression coefficients from the regression models for life satisfaction and positive affect was performed assuming random effects in R (MAc package; http://cran.r-project.org/web/packages/MAc/index.html). This produced an overall effect size and standard error. A false discovery rate correction (Benjamini and Hochberg method) to an alpha level of 0.05 was applied to each of the meta-analyses and to the analysis of general wellbeing. Cohort estimate heterogeneity was assessed by Cochran’s *Q*, which uses the sum of squared deviations of each study’s effect size from the meta-analytic estimate to determine significance. A supplementary meta-analysis was performed on combined life satisfaction, positive affect and general wellbeing measures to obtain a maximal sample size (∼10,000 more individuals than the positive affect analysis). Where a cohort had two measures, the measure with the larger sample was chosen.

#### Extraversion and neuroticism polygenic prediction in UK Biobank

Five polygenic scores were calculated for extraversion and neuroticism based on the significance value for each SNP from the largest respective GWA meta-analysis of these traits (*p* < .01, *p* < .05, *p* < .1, *p* < .5, and *p* < 1 (de Moor et al., 2015; van den Berg et al., 2015). Both GWA studies were based on the same 29 meta-analysis samples that included 63,030 individuals for extraversion and 63,661 individuals for neuroticism. Because there was variation in the personality scale used across samples, an IRT procedure was used to harmonize the personality traits prior to GWA (van den Berg et al., 2014). Polygenic scores (as described in the previous section) for extraversion and neuroticism were created using PRSice software (Euesden et al., 2015) at the five SNP inclusion levels. Before calculating the scores, exclusions were made of SNPs with low minor allele frequency (<0.01) and of SNPs in linkage disequilibrium (*r*^2^ > 0.25) using a clumping method within a 250 kb window. A lower minor allele frequency level exclusion was set for this sample due to its much larger size than the samples comprising the meta-analysis described above; and given the increased reliability of individual effects from the larger GWA meta-analysis, the clumping procedure, which preferentially selects SNPs showing the greatest association, was preferred. For extraversion, the polygenic scores were the composite of 4,271, 18,606, 34,981, 143,525, and 238,487 SNPs for respective *p* < .01, *p* < .05, *p* < .1, *p* < .5, and *p* < 1 inclusion thresholds. For neuroticism, the polygenic scores were the composite of 4,266, 18,427, 34,700, 143,520, and 205,751 SNPs for respective *p* < .01, *p* < .05, *p* < .1, *p* < .5, and *p* < 1 inclusion thresholds. The regression models for polygenic extraversion and neuroticism scores predicting wellbeing included additional independent variables: age at survey, sex, genotyping batch and array, assessment center, and the first 10 genetic principal components (to correct for population stratification). FDR correction was applied to these analyses.

#### Genetic correlations between neuroticism and wellbeing in UK Biobank

Given the large size of UK biobank and the availability of neuroticism and two wellbeing measures, genetic correlations were derived using SNP-based methods (bivariate REML; Lee et al., 2012). This method uses a standard bivariate linear model in which random polygenic effects are fitted and the variance covariance matrix conditioned by a genomic similarity relationship matrix that is estimated from genome-wide SNP information. The program GCTA (Yang et al., 2011) was used for this analysis on unrelated individuals only (individuals with a genetic similarity >0.025 were removed) to remove potential confounding from environmental influences. Observed genotypes were used excluding SNPs with a minor allele frequency less than 0.01. All phenotypes were regressed for the effects of age, sex, assessment center, genetic batch, genetic array, and 10 population stratification components; resulting residual scores were used in the GCTA analysis.

## Results

### NEO-FFI Polygenic Prediction in 14 Cohorts

Meta-analysis results for univariate regression models where personality polygenic scores predict life satisfaction and positive affect can be found in Tables 1 and 2, respectively. These tables display the regression beta, standard error and *p* value for each personality domain at each of the polygenic score inclusion thresholds (i.e., *p* < .01, *p* < .05, *p* < .1, *p* < .5, and *p* < 1).

No tests were significant for life satisfaction or positive affect at the false discovery rate corrected alpha (*q* = 0.002). For positive affect, heterogeneity between cohorts was observed for all neuroticism polygenic scores, four of the extraversion polygenic score estimates and three of the agreeableness polygenic scores (see Supplementary Table S3, for individual cohort betas). The correlations between personality polygenic scores and wellbeing (and corresponding *p* values) are shown in Table 3. In this analysis, no correlations surpassed the FDR corrected significance level. Results from the meta-analysis in which all measures were combined are presented in Supplementary Table S4. No regression coefficients differed significantly from zero and there was significant heterogeneity between cohort estimates for five tests (neuroticism at SNP inclusion *p* < .01, extraversion at SNP inclusion *p* < .5 and *p* < 1, and agreeableness at SNP inclusion *p* < 0.5 and *p* < 1).

### IRT Extraversion and Neuroticism Polygenic Prediction in UK Biobank

**FIGURE 1:**
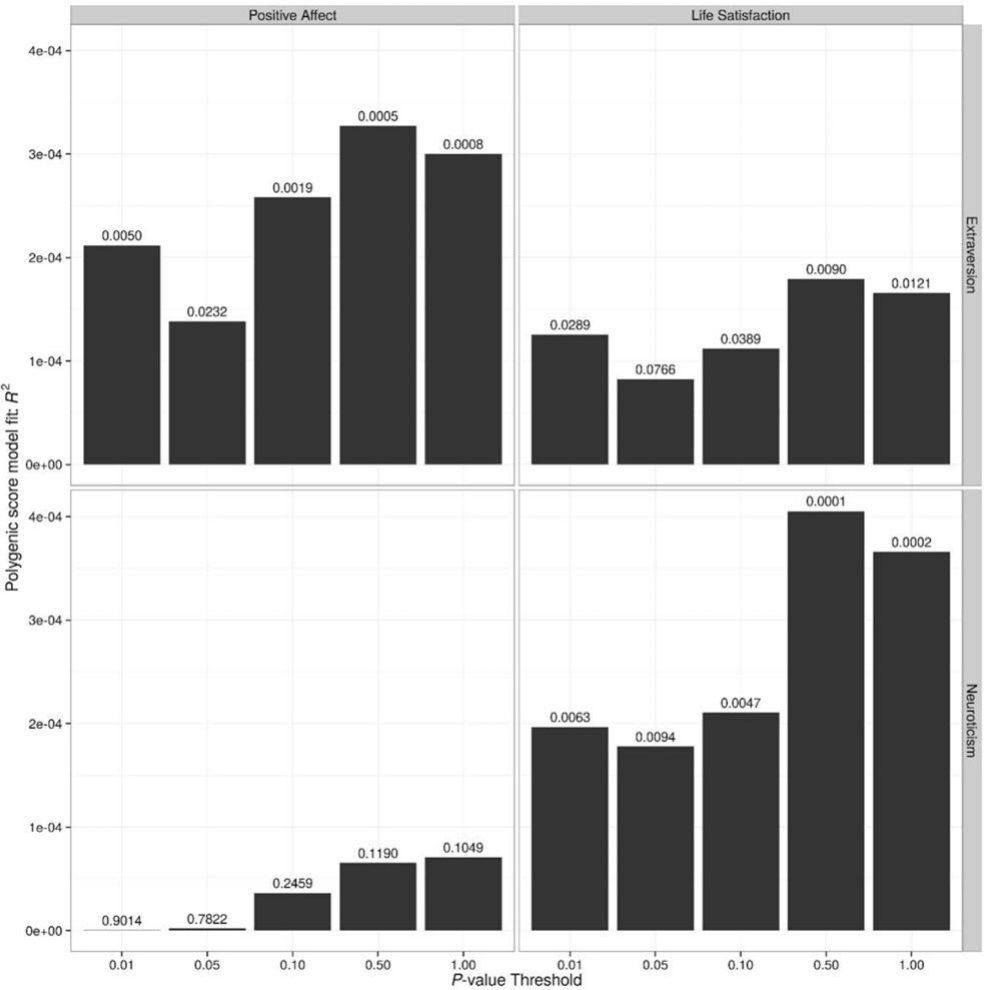
Neuroticism and extraversion polygenic scores at five SNP inclusion thresholds (*x*-axis) predicting life satisfaction and positive affect in UK Biobank. Amount of variance explained by the polygenic scores is depicted on the *y*-axis and the significance value of the polygenic predictor is displayed on the bars.

The significance value and amount of variance explained by the polygenic extraversion and neuroticism scores in predicting life satisfaction and positive affect are shown in Figure 1. The FDR significance level was 0.0325. Extraversion polygenic scores significantly predicted both wellbeing measures (at all SNP inclusion thresholds for positive affect and at three thresholds for life satisfaction), whereas neuroticism polygenic scores significantly predicted only life satisfaction (at all thresholds). In all models, polygenic scores at the more liberal SNP inclusion thresholds explained more variance than the more restrictive SNP inclusion sets. The direction of the effect was as predicted with polygenic neuroticism scores negatively related to life satisfaction and extraversion positively related to measures of wellbeing. The amount of variance explained was extremely small, not exceeding 0.04%.

#### Genetic correlations between neuroticism and wellbeing in UK Biobank

For the analysis of neuroticism and positive affect, 30,367 individuals were included. SNP-based heritabilities of 0.15 (*SE* = 0.02) and 0.08 (*SE* = 0.02) were estimated for respective neuroticism and positive affect measures with a genetic correlation of −0.55 (*SE* = 0.09). The analysis of neuroticism and life satisfaction (*N* = 30,494) gave a heritability of 0.13 (*SE* = 0.02) for life satisfaction and a genetic correlation of −0.49 (*SE* = 0.07) with neuroticism.

## Discussion

These results build upon biometric research showing that common genes influence personality and happiness. The polygenic prediction based on the larger GWA of IRT-based extraversion and neuroticism showed significant association with wellbeing measures at a corrected false discovery rate. The personality polygenic prediction of wellbeing based on the smaller GWA of personality was non-significant for all five NEO-FFI domains. In the NEO-FFI meta-analysis heterogeneity was evident in, at most, four cohorts, suggesting that there were few differences owing to study specific factors (e.g., variation in measurement instrument). Because the meta-analysis and UK Biobank prediction samples were of comparable size (and resulting power), the limiting factor then for these analyses was the difference in power between the GWA studies of the NEO-FFI traits and IRT-based extraversion and neuroticism, on which the polygenic scores were based. In our test of the genetic correlation between neuroticism and wellbeing measures using genetic relationships based on genome-wide SNP data, we found a moderate degree of genetic overlap for both positive affect and life satisfaction.

The finding in UK Biobank that extraversion polygenic scores predicted both life satisfaction and positive affect (measures showing a 0.62 phenotypic correlation in our sample) but that neuroticism polygenic scores predicted only life satisfaction was unexpected given that the combined measure of happiness and satisfaction with life used in the recent GWA of wellbeing significantly predicted neuroticism and extraversion (Okbay et al., 2016). Our finding in UK Biobank of similar-sized genetic correlations between neuroticism with positive affect and life satisfaction would also predict that polygenic neuroticism should relate to positive affect. The null finding might point to type 2 error rather than an interpretation that positive and negative affect are not opposite poles of the same dimension (e.g., Russell & Carroll, 1999). It is likely that the SNP-based genetic correlation between extraversion and positive affect will be stronger than for neuroticism, but we were unable to test this here because no other personality traits were collected in UK Biobank.

The amount of variance in the wellbeing measures explained by the polygenic scores was extremely small, less than half a percent. But given that polygenic neuroticism only predicts 0.66% of variance in neuroticism itself (de Moor et al., 2015), our finding is not unexpected. As GWA meta-analysis studies of personality get larger, this effect size should increase; this is demonstrated by the superior reverse prediction of personality from polygenic wellbeing (Okbay et al., 2016). However, given the low estimated SNP-based heritabilities for neuroticism and wellbeing (<0.15 in our study), the limit for variance explained by a polygenic measure will necessarily be small. Twin and family studies show that heritabilities for personality and wellbeing are at least double that of the SNP-based estimates, which only consider the genetic variation due to common variants. Therefore, further gains in prediction might be achieved by investigating rare and/or structural genetic variants. There are no rare variant studies on personality, but in the only study (Power & Pluess, 2015) to estimate the heritability of all the Five-Factor Model domains using genome-wide SNP data (*N* = 5,011), only neuroticism and openness showed significant genetic influences, suggesting that rare variants might be important. With regard to structural variants, preliminary investigations do not show an effect of large copy number variants on personality (Luciano, MacLeod et al., 2012). Additionally, by using an additive composite of personality SNP effects we may have restricted the prediction of wellbeing. Extended twin studies show non-additive genetic effects for extraversion, neuroticism, and conscientiousness (Hahn et al., 2013; Keller et al., 2005), and measures of wellbeing (Bartels & Boomsma, 2009; Hahn et al., 2016). Further studies are therefore needed to confirm whether different personality traits share greater additive or non-additive genetic variance with wellbeing.

Our study confirms that improvements in polygenic score prediction results from larger meta-analysis GWA studies of the predictor trait. However, it should be noted that Middeldorp et al. (2011) used a subsample (*N* = 13,835) of de Moor et al.’s (2012) NEO-FFI GWA study to create polygenic personality scores that predicted major depressive disorder (from neuroticism) and bipolar disorder (from extraversion). Moreover, Luciano, Huffman et al. (2012) predicted depressive symptoms from polygenic neuroticism using a GWA sample that was even smaller. Accepting that their results were not type 1 errors, one must ask why we failed to predict wellbeing here. One possibility is that the genetic correlations between neuroticism and extraversion are stronger with major depressive disorder (∼0.72; Middeldorp et al., 2005) and bipolar disorder (0.44; Hare et al., 2012) than the genetic correlations between personality and wellbeing (0.20–0.66; Weiss et al., 2008). These estimates, however, are based on twin studies where the similarity across all types of genetic variation is considered. The polygenic scores focus only on common variants, so genetic correlations based on these are more relevant. Using GWA results to estimate genetic correlations, neuroticism showed the same absolute correlation (0.75) with wellbeing (combined positive affect and life satisfaction) and depression (Okbay et al., 2016), although in our bivariate SNP-based method using raw genotypes, genetic correlations between neuroticism and separate positive affect and life satisfaction measures were lower (−0.55 and −0.49). Genetic correlations between extraversion and wellbeing using genome-wide SNP data will be informative. It may well be that personality has stronger genetic links with mental illness than wellbeing. That wellbeing is influenced predominantly by environmental factors unrelated to personality (Weiss et al., 2008) might also limit polygenic prediction.

Using the largest GWA studies to date of extraversion and neuroticism (independent of the UK Biobank sample) we confirmed that polygenic effects for these personality domains influenced wellbeing. Prediction tended to be better when using all SNP data rather than limiting prediction to a smaller number of SNPs with larger effects on personality. This suggests that many genes of very small effect are important for extraversion, neuroticism, and wellbeing. Although neuroticism has captured the interest of many researchers in cognitive psychology and psychiatry, our study also shows an important role of extraversion in mental wellbeing. We expect that genes influencing agreeableness, conscientiousness and openness will also have some role in explaining wellbeing, but our analysis could not reliably address this.

## Supplementary Material

Suppl Mater 1

Suppl Mater 2

Suppl Mater 3

## Figures and Tables

**TABLE 1:** Meta-Analysis Results (Regression Coefficient, Standard Error, *p* Value) for Univariate Analyses of Personality Polygenic Scores (at Five SNP Inclusion Thresholds) Predicting Life Satisfaction (Total *N* = 19,270)

	*p* < .01	*p* < .05	*p* < .1	*p* < .5	*p* < 1
Neuroticism	0.01 (0.007)	0.01 (0.010)	0.016 (0.011)	−0.001 (0.007)	0.002 (0.007)
	*p* = .16	*p* = .27	*p* = .15	*p* = .70	*p* = .66
Extraversion	0.014 (0.010)	0.012 (0.007)	0.015 (0.007)	0.012 (0.007)	0.009 (0.007)
	*p* = .15	*p* = .08	*p* = .031	*p* = .09	*p* = .21
Openness	−0.012 (0.010)	−0.014 (0.01)	−0.014 (0.009)	−0.012 (0.007)	−0.012 (0.007)
	*p* = .25	*p* = .14	*p* = .12	*p* = .08	*p* = .09
Agreeableness	−0.008 (0.007)	0 (0.008)	0.002 (0.007)	0.004 (0.008)	0.006 (0.009)
	*p* = .28	*p* = .75	*p* = .62	*p* = .56	*p* = .46
Conscientiousness	0.004 (0.007)	0.01 (0.007)	0.002 (0.007)	0.017 (0.007)	0.015 (0.007)
	*p* = .51	*p* = .16	*p* = .63	*p* = .021	*p* = .042

Note: False discovery rate *q* = 0.002.

**TABLE 2:** Meta-Analysis Results (Regression Coefficient, Standard Error, *p* Value) for Univariate Analyses of Personality Polygenic Scores (at Five SNP Inclusion Thresholds) Predicting Positive Affect (Total *N* = 46,508)

	*p* < .01	*p* < .05	*p* < .1	*p* < .5	*p* < 1
Neuroticism	−0.006 (0.011)^b^	−0.007 (0.013)^b^	−0.01 (0.014)^b^	−0.019 (0.016)^b^	−0.013 (0.016)^b^
	*p* = .52	*p* = .51	*p* = .43	*p* = .22	*p* = .37
Extraversion	0.001 (0.005)	0.012 (0.008)^a^	0.015 (0.009)^a^	0.02 (0.010)^b^	0.019 (0.010)^b^
	*p* = .68	*p* = .10	*p* = .08	*p* = .048	*p* = .047
Openness	−0.006 (0.005)	−0.001 (0.005)	0.000 (0.005)	−0.004 (0.005)	−0.003 (0.005)
	*p* = .17	*p* = .66	*p* = .73	*p* = .39	*p* = .45
Agreeableness	0.012 (0.006)	0.02 (0.007)^a^	0.02 (0.007)	0.020 (0.009)^b^	0.021 (0.009)^a^
	*p* = .033	*p* = .006	*p* = .004	*p* = .029	*p* = .019
Conscientiousness	0.004 (0.005)	0.005 (0.005)	0.003 (0.005)	0.002 (0.005)	0.000 (0.005)
	*p* = .38	*p* = .24	*p* = .50	*p* = .60	*p* = .74

Note: False discovery rate *q* = 0.002.

a Significant heterogeneity *p* < .05.

b significant heterogeneity *p* < .001.

**TABLE 3:** Correlation and *p* Value for Univariate Analyses of Personality Polygenic Scores (at Five SNP Inclusion Thresholds) Predicting General Wellbeing in the MCTFR (*N* = 6,960)

	*p* < .01	*p* < .05	*p* < .1	*p* < .5	*p* < 1
Neuroticism	−0.009	−0.017	−0.018	−0.023	−0.026
	*p* = .42	*p* = .15	*p* = .13	*p* = .05	*p* = .03
Extraversion	0.011	0.022	0.02	0.015	0.015
	*p* = .35	*p* = .06	*p* = .09	*p* = .21	*p* = .22
Openness	0.011	0.002	0.003	0.01	0.01
	*p* = .34	*p* = .86	*p* = .82	*p* = .39	*p* = .42
Agreeableness	−0.015	0.002	0.008	0.008	0.007
	*p* = .22	*p* = .87	*p* = .53	*p* = .49	*p* = .55
Conscientiousness	−0.011	−0.004	0.012	0.007	0.001
	*p* = .34	*p* = .73	*p* = .31	*p* = .54	*p* = .69

Note: False discovery rate *q* = 0.002.
