# Supplementary material for: Personality Polygenes, Positive Affect, and Life Satisfaction
Source: Twin Res Hum Genet. Author manuscript; Available in PMC 2017 Oct 1. (PMC5125297; doi:10.1017/thg.2016.65)
Supplement: Suppl Mater 1 [file NIHMS829602-supplement-Suppl_Mater_1.docx]

**Supplementary Materials and Method**

**The Austrian Stroke Prevention Study (ASPS).** The ASPS study is a single center prospective follow-up study on the effects of vascular risk factors on brain structure and function in the normal elderly population of the city of Graz, Austria. The procedure of recruitment and diagnostic work-up of study participants has been described previously (Schmidt et al., 1999). A total of 2007 participants were randomly selected from the official community register stratified by gender and 5 year age groups. Individuals were excluded if they had a history of neuropsychiatric disease, including previous stroke, transient ischemic attacks, and dementia, or an abnormal neurologic examination determined on the basis of a structured clinical interview and a physical and neurologic examination. During 2 study periods between September 1991 and March 1994 and between January 1999 and December 2003 an extended diagnostic work-up including neuropsychological testing was done in 1076 individuals aged 45 to 85 years randomly selected from the entire cohort: 509 from the first period and 567 from the second. In 1992, blood was drawn from all study participants for DNA extraction. Genotyping was performed in 996 participants, and 796 who passed genotyping quality control and completed the self-report questionnaire on (elevated) mood (Eigenschaftswörterliste [EWL]) were available for these analyses.

**Health and Retirement Study (HRS).** The HRS is a longitudinal survey of a representative sample of more than 26,000 Americans aged over 50 years (Sonnega et al., 2014) from 17,000 households. The study interviews respondents every two years about income and wealth, health and use of health services, work and retirement, and family connections. DNA was extracted from saliva collected during a face-to-face interview in the respondents' homes. These data represent respondents who provided DNA samples and signed consent forms in 2006, 2008, and 2010. The sample includes 9942 European Americans (n = 4203 males, 5739 females) who were measured on life satisfaction and positive affect. Life satisfaction was measured using the Satisfaction with Life Scale (SWLS) consisting of five items and responses were given on a 6-point scale. The positive affect measure differs across waves. In 2006, it was measured using eight affect questions (e.g., “During the past 30 days, how much of the time did you feel...extremely happy?”) from the Midlife Development Inventory (MIDI). Responses were given on a 5-point scale. In 2008, 2010 and 2012, it was measured using thirteen questions (5-point scale). Eleven of these questions were obtained from Positive and Negative Affect Schedule—Expanded Form (PANAS-X). At the time of data collection, participants ranged in age from 30 to 107 years (mean age was 67.21 (SD = 10.98) years).

**Lifelines.** Individuals were included from the LifeLines study, a multidisciplinary prospective population-based cohort study, examining health and health-related behaviors of persons living in the Northern region of the Netherlands (Scholtens et al., 2015). Genotype and phenotype data were available from 1051 participants who responded to the question “Are you basically satisfied with your life?” (adapted from the World Values Survey 1981 - <http://www.worldvaluessurvey.org/wvs.jsp>). Responses are on a 5-points scale ranging from 1 to 5. Participants ranged in age from 26 to 88 years; their mean age was 69.43 (SD = 5.7). The mean score of the questionnaire was 4.1. For positive affect, the ten positive items adapted from the PANAS were used and available in 11,971 individuals (during the past 30 days indicate how much of the time you feel: 1. Interested; 2. Excited; 3. Strong; 4. Enthusiastic; 5. Proud; 6. Alert; 7. Inspired; 8. Determined; 9. Attentive; 10. Active). Responses are given on a 5-point scale and averaged scores were used only for individuals with complete data. Participants ranged in age from 18 to 88 years; the mean age of participants was 47.74 years (SD = 11.1). The mean PANAS score was 3.5 (the maximum score possible was 5).

**Lothian Birth Cohort of 1921 (LBC1921).** This sample comprised relatively healthy people living in the Lothian region of Scotland (Deary et al., 2012). Participants were born in 1921 and from the age of 79 years they were assessed on psychological and medical traits, including the SWLS Scale. Genotype and phenotype data were available for 447 participants of whom 57.5% were female; mean age of 81.2 years (SD = 0.28; range= 80.7 to 81.7). The range of life satisfaction scores was 5 to 35 with a mean of 25.4 (SD = 6.1).

**Minnesota Center for Twin and Family Research (MCTFR).** Data from the MCTFR were collected as part of two different longitudinal studies, the Minnesota Twin Family Study (MTFS) and the Sibling Interaction and Behavior Study (SIBS). The MTFS is a study of reared-together, same-sex twins and their parents, and the SIBS is a study of families of different types (some include adopted offspring). Parents and offspring were measured at baseline on affective and cognitive dimensions of wellbeing using the Multidimensional Personality Questionnaire. The range of scores was between 21 and 72 (mean = 55.53; SD = 7.84) with higher scores representing higher wellbeing. Data were available for up to 5 follow-ups for offspring in the MTFS and up to 2 for offspring in the SIBS. We selected data from the first assessment with complete data for each individual. DNA was obtained primarily from blood sample (otherwise, saliva). The total sample with MPQ data included 9071 participants (53% female), and of those participants, 6971 had both wellbeing scores and usable genotyping data (seeMiller et al., 2012). The final sample included 3416 males and 3555 females within 2315 families (age mean = 34.61, SD = 11.15, range = 13.63 – 64.65).

**Netherlands Twin Register (NTR).** Participants were registered with the NTR, established by the department of Biological Psychology at the VU University Amsterdam (van Beijsterveldt et al., 2013; Willemsen et al., 2013). Only NTR participants who did not participate in the de Moor and colleagues’ original GWAS of the NEO-FFI domains (de Moor et al., 2012) were selected. Genotypic and phenotypic information were present in 3419 (positive affect) and 4549 (life satisfaction) of these participants. Mean age for positive affect was 28.4 years (10-80) and for life satisfaction, 36.7 years (10-90). Life satisfaction was measured longitudinally using the Satisfaction with Life Scale. Positive affect was measured longitudinally using four questions that were adapted from the Subjective Happiness Scale (e.g. “On the whole, I am a happy person”) with responses on a 7-point scale, resulting in a minimum score of 4 and a maximum score of 28.

**QIMR Berghofer Queensland Institute of Medical Research (QIMRB).** Wellbeing data were collected in two twin family studies (NAGS and TWIN 89) conducted at the QIMRB. Both studies consisted of posted questionnaires assessing health and lifestyle issues as well as demographic information. The first study was conducted between 1996 and 2000 (Mosing et al., 2009) and the second between 2001 and 2005 (Hansell et al., 2008). A single item assessed on a four point scale was used to measure positive affect: “How would you describe your emotional wellbeing?” 1=Poor (n = 40), 2=Fair (n = 303), 3=Good (n = 1215), and 4=Excellent (n = 749). The data contained 2307 individuals, consisting of 1190 females and 1117 males, ranging between 19 to 84 years of age (mean 43.7 and SD = 10.39).

**Rotterdam Study (RS).** The RS is a prospective cohort study ongoing since 1990 in the city of Rotterdam in The Netherlands (Hofman et al., 2013). The study targets cardiovascular, endocrine, hepatic, neurological, ophthalmic, psychiatric, dermatological, otolaryngological, locomotor, and respiratory diseases. As of 2008, 14,926 participants aged 45 years or over comprise the Rotterdam Study cohort. Positive affect was obtained by taking the average of four positively framed items in the Center for Epidemiological Studies Depression (CES-D) Scale for a total of 8918 individuals in the three Rotterdam Study sub-cohorts: RS-I, RS-II and RS-III. There were 3888 individuals with non-missing phenotype in RS-I (59% women), 2066 in RS-II (54% women), and 2964 in RS-III (56% women). The mean age was 66.2 years in the RS-I sample (SD = 7.2), 64.6 in RS-II (SD = 7.8), 57.0 in RS-III (SD = 6.7). The data were collected between 1997 and 2008.

**The Rush Memory and Aging Project (MAP).** These participants were from an ongoing clinical pathologic studies of aging and dementia. MAP began in 1997 and includes participants aged 55 or over without known dementia at enrolments (Bennett et al., 2012). The participants were from retirement communities or individual homes in north-eastern Illinois and agreed to annual clinical evaluation and organ donation. A total of 351 participants had complete data on the SWLS and were included in the analysis. The mean age was 83.9 (SD = 6.5) years, and 83 (23.7%) of the participants were male.

**Swedish Twin Registry (STR).** The STR is a large, population-based twin registry. Between 1998 and 2002 STR administered a survey called the Screening Across the Lifespan Twin Study (SALT) to twins born between 1911 and 1958. Positive affect was assessed using the “happy” item from the CES-D included in SALT. A subsample of SALT was genotyped in the TwinGene study (Magnusson et al., 2013) using DNA from 9896 individual subjects (all available dizygotic twins plus one twin from each available monozygotic twin pair). After quality control there were 9617 individuals remaining. Three hundred and twenty-two individuals were dropped due to missing age and sex values. Selecting one twin per family resulted in 6680 (52% women) individuals, on which the analyses were conducted; their mean birth year was 1941 (SD = 8.9) with an age range of 51 to 98 years (mean = 67.6, SD = 8.91).

**TRacking Adolescents' Individual Lives Survey (TRAILS)**. This is a prospective cohort study of Dutch adolescents with bi- or triennial measurements from age 11 to adulthood (see (Oldehinkel et al., 2015). Five assessment waves have been completed to date, which ran from March 2001 to July 2002 (T1), September 2003 to December 2004 (T2), September 2005 to August 2007 (T3), October 2008 to September 2010 (T4), and January 2012 to December 2013 (T5). Data for the present study were collected during T4. At T1, 2230 (pre)adolescents were enrolled in the study (response rate 76%, mean age = 11.1 (SD = 0.6), 51% girls(de Winter et al., 2005)(de Winter et al., 2005)(de Winter et al., 2005)([de Winter et al., 2005](#_ENREF_6)), of whom 84% (n = 1816, mean age 16.3 (SD = 0.7), 52% girls) participated at T4; 1214 of these (mean age = 18.5 (SD = 0.6) had available genotyping. Life satisfaction and positive affect were each measured by the single respective items, ‘How satisfied are you, altogether, with your present life, on a scale from 1 to 10?’ (mean = 7.5, SD=1.49) and ‘How happy are you, altogether, on a scale from 1 to 10?’ (mean = 7.56, SD=1.43). Participating centers of TRAILS include the University Medical Center and University of Groningen, the Erasmus University Medical Center Rotterdam, the University of Utrecht, the Radboud Medical Center Nijmegen, and the Parnassia Bavo group, all in the Netherlands.

**The Young Finns Study (YFS).** The YFS is an ongoing, population-based prospective cohort study started in 1980 with a baseline sample of 3596 children and adolescents derived from six birth cohorts aged 3, 6, 9, 12, 15, and 18 years at baseline (http://youngfinnsstudy.utu.fi/). Participants were recruited from five areas according to locations of university cities with a medical school (Helsinki, Kuopio, Oulu, Tampere and Turku). In each location, individuals from urban and surrounding rural areas were selected at random from the population registry (based on their unique personal social security number) to be invited in the study. Data on positive affect data were collected with a brief 5-item version of the PANAS questionnaire from 1709 participants in 2012. The PANAS included the following 5 positive affect items: Alert, Excited, Determined, Attentive, and Active. Life satisfaction was measured using an average of 3 items: satisfaction as a parent (“I am satisfied with myself as a parent”), as a spouse (“I am satisfied with myself as a spouse”), and as a worker. Each item was rated on a 5-point scale (1=dissatisfied, 5=satisfied). The mean age of participants was 41.8 years (SD = 5.1) at the time of assessment, and 1,006 participants (58.9%) were female.

**References**

Bennett, D. A., Schneider, J. A., Buchman, A. S., Barnes, L. L., Boyle, P. A., & Wilson, R. S. (2012). Overview and Findings from the Rush Memory and Aging Project. *Current Alzheimer Research, 9*(6), 646-663.

de Moor, M. H., Costa, P. T., Terracciano, A., Krueger, R. F., de Geus, E. J., Toshiko, T., et al. (2012). Meta-analysis of genome-wide association studies for personality. *Mol Psychiatry, 17*(3), 337-349.

de Winter, A. F., Oldehinkel, A. J., Veenstra, R., Brunnekreef, J. A., Verhulst, F. C., & Ormel, J. (2005). Evaluation of non-response bias in mental health determinants and outcomes in a large sample of pre-adolescents. *European Journal of Epidemiology, 20*(2), 173-181.

Deary, I. J., Gow, A. J., Pattie, A., & Starr, J. M. (2012). Cohort Profile: The Lothian Birth Cohorts of 1921 and 1936. *International Journal of Epidemiology, 41*(6), 1576-1584.

Hansell, N. K., Agrawal, A., Whitfield, J. B., Morley, K. I., Zhu, G., Lind, P. A., et al. (2008). Long-term stability and heritability of telephone interview measures of alcohol consumption and dependence. *Twin Res Hum Genet, 11*(3), 287-305.

Hofman, A., Darwish Murad, S., van Duijn, C. M., Franco, O. H., Goedegebure, A., Ikram, M. A., et al. (2013). The Rotterdam Study: 2014 objectives and design update. *Eur J Epidemiol, 28*(11), 889-926.

Miller, M. B., Basu, S., Cunningham, J., Eskin, E., Malone, S. M., Oetting, W. S., et al. (2012). The Minnesota Center for Twin and Family Research Genome-Wide Association Study. *Twin Research and Human Genetics, 15*(6), 767-774.

Mosing, M. A., Gordon, S. D., Medland, S. E., Statham, D. J., Nelson, E. C., Heath, A. C., et al. (2009). Genetic and environmental influences on the co-morbidity between depression, panic disorder, agoraphobia, and social phobia: a twin study. *Depress Anxiety, 26*(11), 1004-1011.

Oldehinkel, A. J., Rosmalen, J. G. M., Buitelaar, J. K., Hoek, H., Ormel, J., Raven, D., et al. (2015). Cohort Profile Update: The TRacking Adolescents' Individual Lives Survey (TRAILS). *International Journal of Epidemiology, 44*(1), 76-U94.

Schmidt, R., Fazekas, F., Kapeller, P., Schmidt, H., & Hartung, H. P. (1999). MRI white matter hyperintensities - Three-year follow-up of the Austrian stroke prevention study. *Neurology, 53*(1), 132-139.

Scholtens, S., Smidt, N., Swertz, M. A., Bakker, S. J., Dotinga, A., Vonk, J. M., et al. (2015). Cohort Profile: LifeLines, a three-generation cohort study and biobank. *Int J Epidemiol, 44*(4), 1172-1180.

Sonnega, A., Faul, J. D., Ofstedal, M. B., Langa, K. M., Phillips, J. W., & Weir, D. R. (2014). Cohort Profile: the Health and Retirement Study (HRS). *Int J Epidemiol, 43*(2), 576-585.

van Beijsterveldt, C. E., Groen-Blokhuis, M., Hottenga, J. J., Franic, S., Hudziak, J. J., Lamb, D., et al. (2013). The Young Netherlands Twin Register (YNTR): longitudinal twin and family studies in over 70,000 children. *Twin Res Hum Genet, 16*(1), 252-267.

Willemsen, G., Vink, J. M., Abdellaoui, A., den Braber, A., van Beek, J. H., Draisma, H. H., et al. (2013). The Adult Netherlands Twin Register: twenty-five years of survey and biological data collection. *Twin Res Hum Genet, 16*(1), 271-281.
