# Supplementary material for: Personality Polygenes, Positive Affect, and Life Satisfaction
Source: Twin Res Hum Genet. Author manuscript; Available in PMC 2017 Oct 1. (PMC5125297; doi:10.1017/thg.2016.65)
Supplement: Suppl Mater 2 [file NIHMS829602-supplement-Suppl_Mater_2.docx]

**Supplementary Lifelines Author List**

Behrooz Z Alizadeh (1), H Marike Boezen (1), Lude Franke (2), Pim van der Harst (3), Gerjan Navis (4), Marianne Rots (5), Harold Snieder (1), Ronald P Stolk (1,6), Morris Swertz (2), Bruce HR Wolffenbuttel (7), Cisca Wijmenga (2)

(1) Department of Epidemiology, University of Groningen, University Medical Center Groningen, The Netherlands

(2) Department of Genetics, University of Groningen, University Medical Center Groningen, The Netherlands

(3) Department of Cardiology, University of Groningen, University Medical Center Groningen, The Netherlands

(4) Department of Internal Medicine, Division of Nephrology, University of Groningen, University Medical Center Groningen, The Netherlands

(5) Department of Medical Biology, University of Groningen, University Medical Center Groningen, The Netherlands

(6) LifeLines Cohort Study, University of Groningen, University Medical Center Groningen, The Netherlands

(7) Department of Endocrinology, University of Groningen, University Medical Center Groningen, The Netherlands

**Supplementary Acknowledgements**

**ASPS:** The authors thank the staff and the participants for their valuable contributions. We thank Birgit Reinhart for her long-term administrative commitment, Elfi Hofer for the technical assistance at creating the DNA bank, Ing. Johann Semmler and Anita Harb for DNA sequencing and DNA analyses by TaqMan assays and Irmgard Poelzl for supervising the quality management processes after ISO9001 at the biobanking and DNA analyses. **HRS**: Our genotyping was conducted by the NIH Center for Inherited Disease Research (CIDR) at Johns Hopkins University. Genotyping quality control and final preparation of the data were performed by the Genetics Coordinating Center at the University of Washington. **LBC1921:** We thank the LBC1921 participants and team members. **LifeLines Cohort Study**: We thank Behrooz Alizadeh, Annemieke Boesjes, Marcel Bruinenberg, Noortje Festen, Pim van der Harst, Ilja Nolte, Lude Franke, Mitra Valimohammadi for their help in creating the GWAS database, and Rob Bieringa, Joost Keers, René Oostergo, Rosalie Visser, Judith Vonk for their work related to data-collection and validation. The authors are grateful to the study participants, the staff from the LifeLines Cohort Study and the contributing research centers delivering data to LifeLines and the participating general practitioners and pharmacists. **NTR (Netherlands Twin Register) -** We would like to thank all the twins and family members for their participation. For access to NTR results, please contact Meike Bartels at [m.bartels@vu.nl](mailto:m.bartels@vu.nl). **QIMRB**: We would like to thank the twins and their families registered at the ATR for their participation and QIMRB for sample collection. **RS:** The authors are grateful to the study participants, the staff from the Rotterdam Study and the participating general practitioners and pharmacists. Researchers who wish to use data of the Rotterdam Study must obtain approval from the Rotterdam Study Management Team. They are advised to contact the PI of the Rotterdam Study, Dr Albert Hofman ([a.hofman@erasmusmc.nl](mailto:a.hofman@erasmusmc.nl)). **STR:** Researchers interested in using STR data must obtain approval from the Swedish Ethical Review Board and from the Steering Committee of the Swedish Twin Registry. Researchers using the data are required to follow the terms of an Assistance Agreement containing a number of clauses designed to ensure protection of privacy and compliance with relevant laws. For Further information, contact Patrik Magnusson ([Patrik.magnusson@ki.se](mailto:Patrik.magnusson@ki.se)). **The Rush Memory and Aging Project** We thank the study participants and the staff of the Rush Alzheimer’s Disease Center. **UK Biobank:** This research has been conducted using the UK Biobank Resource. **YFS**: The expert technical assistance in statistical analysis by Irina Lisinen, Mika Helminen, and Ville Aalto is gratefully acknowledged.
