# Supplementary material for: Personality Polygenes, Positive Affect, and Life Satisfaction
Source: Twin Res Hum Genet. Author manuscript; Available in PMC 2017 Oct 1. (PMC5125297; doi:10.1017/thg.2016.65)
Supplement: Suppl Mater 3 [file NIHMS829602-supplement-Suppl_Mater_3.docx]

S1 Table. Cohort Characteristics, including Details of Wellbeing Phenotype and of Genetic Data used for Polygenic Score Calculation.

| Cohort | n | Sample Characteristics (ethnicity, range, mean, SD) | Phenotype | Genotyping Chip | Sample call rate exclusion filter | Polygenic Scores: Observed vs Imputed | Number of SNPs after LD pruning |
| --- | --- | --- | --- | --- | --- | --- | --- |
| ASPS | 796 | European; 45-85 years, 65.1 (SD 7.89) | “How do you feel at the moment” item sampling 11 descriptors from EWL (Janke WD, 1977) [PA] | Illumina Human610-Quad BeadChip | ≤0.98 | HapMap CEU, Phase II, release 22, build 36 | 116,818 |
| HRS | 9938 [LS]; 9117 [PA] | European Americans; 30-107, 67.21 (SD 10.98) years | Satisfaction with Life Scale (Diener, Emmons, Larsen, & Griffin, 1985) [LS]; MIDI affect scales (Mroczek & Kolarz, 1998), PANAS-X (David Watson & Clark, 1999) [PA] | HumanOmni2.5-4v1 and HumanOmni2.5-8v1 | <0.98 | Observed | 166,084 |
| Lifelines | 1051 [LS]  11,971 [PA] | Dutch; 26-88, 69.4 (SD 5.7) years  Dutch; 18-88, 47.7 (SD 11.7) years | “Are you basically satisfied with your life?” on a 5-point scale [LS]  PANAS (10 items) (D. Watson, Clark, & Tellegen, 1988) [PA] | Illumina BeadScanner, Beagle 3.1.0 (Dec 2009) | <0.95 | 1000G b37 | 123,704 |
| LBC1921 | 447 | Scottish; 80.7-81.7, 81.2 (SD 0.28) years | Satisfaction with Life Scale (Diener, et al., 1985) [LS] | Illumina Human 610_Quadv1 | ≤0.95 | HapMap CEU,  Phase II, release 22, build 36 | 122,973 |
| MAP | 351 | ≥ 55; 83.9 (SD 6.5) years | Satisfaction with Life Scale (Diener, et al., 1985) [LS] | Affymetrix Genechip 6.0 | ≤0.95 | HapMap CEU,  Phase II, release 22, build 36 | 133,100 |
| MCTFR | 6960 | European ancestry; 13-64, 34.6 (SD 11.5) years | Wellbeing scale from the Multidimensional Personality Questionnaire (Tellegen & Waller, 2008) [GW] | Illumina Human660W-Quad | <0.99 | HapMap CEU,  Phase II, release 22, build 36 | 118,142 |
| NTR | 4549 [LS]; 3417 [PA] | Dutch; 10-90, 36.7 (SD 17.4) years [LS]; 10-80, 28.4 (SD 14.9) years [PA] | Satisfaction with Life Scale (Diener, et al., 1985) [LS]; Subjective Happiness Scale (Lyubomirsky & Lepper, 1999) [PA] | Affymetrix 6.0, Perlegen-Affymetrix 5.0, Illumina 660, Illumina 1M, Illumina 370K | <0.95 | 1000G b37 | 253,302 |
| QIMR | 2307 | European ancestry; 19-84, 43.7 (SD 10.39) years | “How would you describe your emotional wellbeing?”  1=Poor, 2=Fair, 3=Good, 4=Excellent [PA] | Illumina HumanCNV370-Quadv3; Illumina Human610-Quadv1; Illumina 317K | ≤0.98 | HapMap CEU, Phase I+II data, release 22, Build 36 | 96,014 |
| RS-I | 3888 | European ancestry; 55-96, 66.2 (SD 7.2) years | 4 positively framed items from the CES-D (Radloff, 1977) [PA] | Illumina HumanHap 550 V.3 | ≤0.98 | Observed | 112,463 |
| RS-II | 2066 | European ancestry; 55-95, 64.6 (SD 7.8) years | 4 positively framed items from the CES-D (Radloff, 1977) [PA] | Illumina HumanHap 550 V.3 DUO; Illumina HumanHap 610 QUAD | ≤0.975 | Observed | 112,368 |
| RS-III | 2964 | European ancestry; 45-89, 57 (SD 6.7) years | 4 positively framed items from the CES-D (Radloff, 1977) [PA] | Illumina HumanHap 610 QUAD | ≤0.975 | Observed | 112,577 |
| STR | 6680 | Swedish; 51-98, 67.56 (SD 8.9) years | ‘Happy’ item from the CES-D (Radloff, 1977) [PA] | Illumina HumanOmniEx press-12v1_A | ≤0.97 | HapMap CEU, Phase II, release 22, build 36 | 120,331 |
| TRAILS | 1214 | Dutch; 17-20, 18.5 (SD 0.60) years | “How satisfied are you, altogether, with your present life?” [LS]; “How happy are you, altogether?” [PA] | Illumina Cyto SNP12 v2 | <0.95 | HapMap CEU,  Phase II, release 22, build 36 | 107,670 |
| YFS | 1738 | Finnish; 35-50, 41.8 (SD 5.1) years | Satisfaction as a parent, spouse and worker [LS]; PANAS (5-items) (D. Watson, et al., 1988) [PA] | Illumina custom made BeadChip Human 670K-Quad | <0.95 | HapMap CEU,  Phase II, release 22, build 36 | 108,898 |

LS: Life Satisfaction; PA: Positive Affect; GW: General wellbeing

S2 Table. The Number of SNPs Included in Each Polygenic Score at the Differing Threshold Inclusion Levels for Each Cohort. A Range is given across the Five Personality Polygenic Scores for each of the Thresholds where Applicable.

| Cohort | p<.01 | p<.05 | p<.1 | p<.5 | p<1 |
| --- | --- | --- | --- | --- | --- |
| ASPS | 1211-1290 | 5906-6070 | 11706-12050 | 58370-58974 | 116818 |
| HRS | 778-872 | 3908-4005 | 7683-7924 | 38495-38996 | 77422 |
| LBC1921 | 1270–1353 | 6197-6348 | 12374-12576 | 61645-62004 | 122973 |
| Lifelines | 1252-1316 | 6211-6431 | 12448-12754 | 61825-62339 | 123697-123704 |
| MAP | 1360-1410 | 6727-6904 | 13322-13757 | 66731-67194 | 133100 |
| MCTFR | 1228-1296 | 5972-6150 | 11694-12157 | 58931-59592 | 118142 |
| NTR | 2727-2854 | 12970-13044 | 25520-26035 | 126300-127820 | 253064-253302 |
| QIMR | 1001-1038 | 4864-4996 | 9560-9827 | 48011-48269 | 96014 |
| RS1 | 1125-1230 | 5691-5940 | 11173-11482 | 56267-56575 | 112463 |
| RS2 | 1140-1258 | 5665-5867 | 11210-11484 | 56298-56644 | 112368 |
| RS3 | 1163-1222 | 5680-5904 | 11208-11470 | 56437-56610 | 112577 |
| STR | 1209-1301 | 6049-6247 | 12012-12459 | 60228-60889 | 120331 |
| TRAILS | 1040-1119 | 5213-5288 | 10352-10504 | 51452-51830 | 102793 |
| YFS | 1129-1204 | 5469-5603 | 10851-11112 | 54454-54995 | 108898 |

S3 Table. Individual Cohort Regression Betas for Polygenic Neuroticism, Extraversion and Agreeableness Scores Predicting Positive Affect, where Heterogeneity was Observed.

|  |  | Neuroticism | | | | | Extraversion | | | | Agreeableness | | |  |  |
| --- | --- | --- | --- | --- | --- | --- | --- | --- | --- | --- | --- | --- | --- | --- | --- |
|  | N | p<.01 | p<.05 | p<.10 | p<.50 | p<1 | p<.05 | p<.10 | p<.50 | p<1 | p<.05 | p<.50 | p<1 | | |
| MAP | 351 | 0.046 | 0.016 | 0.043 | -0.002 | -0.005 | -0.004 | -0.003 | 0.066 | 0.071 | 0.056 | 0.039 | 0.041 | |  |
| STR | 6680 | -0.015 | 0.006 | 0.003 | 0.008 | 0.008 | -0.008 | -0.006 | -0.007 | -0.009 | -0.002 | -0.025^a^ | -0.021 | |  |
| RS1 | 3888 | -0.029 | -0.04 | -0.034 | -0.023 | 0.023 | 0.005 | -0.001 | .000 | 0.003 | 0.014 | 0.006 | 0.004 | |  |
| RS2 | 2066 | -0.011 | -0.007 | 0.003 | -0.001 | 0.002 | 0.013 | 0.002 | 0.023 | 0.026 | 0.03 | 0.06^b^ | 0.05^a^ | |  |
| RS3 | 2964 | -0.003 | -0.019 | -0.029 | -0.02 | -0.02 | 0.04^a^ | 0.04^a^ | 0.03 | 0.03 | 0.013 | 0.024 | 0.026 | |  |
| QIMR | 2307 | -0.09^c^ | -0.126^c^ | -0.151^c^ | -0.178^c^ | -0.18^c^ | 0.064^b^ | 0.083^c^ | 0.109^c^ | 0.109^c^ | 0.062^b^ | 0.069^b^ | 0.071^b^ | |  |
| ASPS | 796 | 0.085^a^ | 0.057 | 0.031 | 0.009 | 0.008 | 0.045 | 0.046 | 0.027 | 0.02 | 0.077^a^ | 0.056 | 0.056 | |  |
| YFS | 1738 | 0.054^a^ | 0.026 | 0.014 | -0.012 | .000 | 0.011 | 0.031 | 0.03 | 0.027 | 0.032 | 0.017 | 0.009 | |  |
| HRS | 9117 | -0.002 | 0.000 | 0.003 | 0.008 | 0.006 | 0.001 | 0.007 | 0.000 | 0.000 | 0.007 | 0.011 | 0.012 | |  |
| TRAILS | 1213 | -0.013 | 0.033 | 0.029 | 0.02 | 0.023 | -0.022 | -0.006 | 0.009 | 0.009 | 0.017 | -0.019 | -0.011 | |  |
| Lifelines | 11971 | 0.007 | -0.005 | 0.004 | -0.001 | -0.002 | -0.01 | -0.016 | -0.016 | -0.009 | -0.005 | 0.001 | 0.005 | |  |
| NTR | 3417 | -0.032 | -0.01 | -0.001 | -0.025 | -0.022 | 0.04 | 0.03 | 0.034 | 0.021 | 0.049^a^ | 0.053^a^ | 0.058^b^ | |  |

^a^ p<.05, ^b^ p<.01, ^c^ p<.0001

S4 Table. Meta-Analysis Results (Regression Beta, Standard Error, P-Value) for Univariate Analyses of Personality Polygenic Scores (At 5 SNP Inclusion Thresholds) Predicting Combined Measures of Life Satisfaction, Positive Affect, and General Wellbeing (Total N = 55,869).

|  | p<.01 | p<.05 | p<.1 | p<.5 | p<1 |
| --- | --- | --- | --- | --- | --- |
| Neuroticism | -.003(.009)^c^  p=.63 | -.01 (.011)  p=.35 | -.011 (.012)  p=.34 | -.019 (.013)  p=.16 | -.014 (.014)  p=.31 |
| Extraversion | .003 (.004)  p=.42 | .013 (.006)  p=.036 | .017 (.007)^a^  p=.027 | .018 (.008)^a^  p=.038 | .018 (.008)^a^  p=.033 |
| Openness | -.003 (.005)  p=.43 | -.003 (.004)  p=.48 | -.002 (.004)  p=.57 | -.002 (.004)  p=.52 | -.002 (.004)  p=.59 |
| Agreeableness | .005 (.006)  p=.41 | .010 (.006)  p=.08 | .012 (.005)  p=.024 | .014 (.007)^b^  p=.051 | .015 (.007)^a^  p=.035 |
| Conscientiousness | -.001 (.004)  p=.62 | .005 (.004)  p=.27 | .005 (.004)  p=.27 | .007 (.004)  p=.09 | .006 (.004)  p=.17 |

FDR q = .002; ^a^ p<.05; ^b^ p< .01; ^c^ p< .001
